# Controversies in the management of advanced prostate cancer

**DOI:** 10.1038/sj.bjc.6690024

**Published:** 1999-09-24

**Authors:** C J Tyrrell

**Affiliations:** Oncology Research Unit, Derriford Hospital, Plymouth, UK

**Keywords:** hormonal therapy, advanced prostate cancer, combined androgen blockade, luteinizing hormone-releasing hormone analogues, surgical castration, anti-androgens

## Abstract

For advanced prostate cancer, the main hormone treatment against which other treatments are assessed is surgical castration. It is simple, safe and effective, however it is not acceptable to all patients. Medical castration by means of luteinizing hormone-releasing hormone (LH-RH) analogues such as goserelin acetate provides an alternative to surgical castration. Diethylstilboestrol, previously the only non-surgical alternative to orchidectomy, is no longer routinely used. Castration reduces serum testosterone by around 90%, but does not affect androgen biosynthesis in the adrenal glands. Addition of an anti-androgen to medical or surgical castration blocks the effect of remaining testosterone on prostate cells and is termed combined androgen blockade (CAB). CAB has now been compared with castration alone (medical and surgical) in numerous clinical trials. Some trials show advantage of CAB over castration, whereas others report no significant difference. The author favours the view that CAB has an advantage over castration. No study has reported that CAB is less effective than castration. Of the anti-androgens which are available for use in CAB, bicalutamide may be associated with a lower incidence of side-effects compared with the other non-steroidal anti-androgens and, in common with nilutamide, has the advantage of once-daily dosing. Only one study has compared anti-androgens within CAB: bicalutamide plus LH-RH analogue and flutamide plus LH-RH analogue. At 160-week follow-up, the groups were equivalent in terms of survival and time to progression. However, bicalutamide caused significantly less diarrhoea than flutamide. Withdrawal and intermittent therapy with anti-androgens extend the range of treatment options. © 1999 Cancer Research Campaign


*British Journal of Cancer* (1999) **79**(1), 146–155
© 1999 Cancer Research Campaign

### Controversies in the management of advanced prostate cancer

**CJ Tyrrell**

Oncology Research Unit, Derriford Hospital, Plymouth, UK

**Summary** For advanced prostate cancer, the main hormone treatment against which other treatments are assessed is surgical castration. It is simple, safe and effective, however it is not acceptable to all patients. Medical castration by means of luteinizing hormone-releasing hormone (LH-RH) analogues such as goserelin acetate provides an alternative to surgical castration. Diethylstilboestrol, previously the only non-surgical alternative to orchidectomy, is no longer routinely used. Castration reduces serum testosterone by around 90%, but does not affect androgen biosynthesis in the adrenal glands. Addition of an anti-androgen to medical or surgical castration blocks the effect of remaining testosterone on prostate cells and is termed combined androgen blockade (CAB). CAB has now been compared with castration alone (medical and surgical) in numerous clinical trials. Some trials show advantage of CAB over castration, whereas others report no significant difference. The author favours the view that CAB has an advantage over castration. No study has reported that CAB is less effective than castration. Of the anti-androgens which are available for use in CAB, bicalutamide may be associated with a lower incidence of side-effects compared with the other non-steroidal anti-androgens and, in common with nilutamide, has the advantage of once-daily dosing. Only one study has compared anti-androgens within CAB: bicalutamide plus LH-RH analogue and flutamide plus LH-RH analogue. At 160-week follow-up, the groups were equivalent in terms of survival and time to progression. However, bicalutamide caused significantly less diarrhoea than flutamide. Withdrawal and intermittent therapy with anti-androgens extend the range of treatment options.

**Keywords:** hormonal therapy; advanced prostate cancer; combined androgen blockade; luteinizing hormone-releasing hormone analogues; surgical castration; anti-androgens

Prostate cancer is one of the most common forms of cancer in men and, after lung cancer, is the most common cause of male cancer death (Figure 1). The incidence of prostate cancer varies around the world and is highest in Western countries such as the USA and Scandinavia (Parker et al, 1997). In the USA, the incidence and prevalence of prostate cancer is increasing, with an estimate (based on deaths from 1979 to 1993) of 334 000 new cases and 41 800 deaths for 1997 (Parker et al, 1997; Wingo et al, 1997). The incidence of prostate cancer in Japan, although low at one-tenth of North America, is also rising rapidly, perhaps because of adoption of a more Westernized lifestyle (Dearnaley, 1994).

Prostate cancer is rarely diagnosed before the age of 50 and the incidence increases markedly between the ages of 60 and 80 years, with a median age at diagnosis of 72 years (Brawley and Kramer, 1994). As the male population over 75 years increases, so too do the number of men at risk from prostate cancer.

Prostate cancer growth is stimulated by androgens, principally testosterone, therefore androgen deprivation is an essential component in the treatment of this disease. Advanced prostate cancer is usually defined as a disease which has become metastatic or locally advanced, and is, therefore, incurable. Traditional treatment for advanced prostate cancer is castration (surgical or medical) which reduces serum testosterone levels by about 90% (Labrie et al, 1985; Lunglmayr et al, 1988). However, castration does not affect androgen biosynthesis in the adrenal glands and addition of an anti-androgen may be used to block the effect of remaining testosterone on the prostate cells. The addition of anti-androgen to castration is commonly known as combined androgen blockade (CAB) or maximal androgen blockade (MAB).

This review will discuss the relative advantages and disadvantages of hormonal treatments available for advanced prostate cancer (Table 1), with particular emphasis on CAB. Related treatment options and additional factors in the selection of treatment will also be reviewed.

### MAIN HORMONAL TREATMENTS AVAILABLE

First-line hormonal treatment of advanced prostate cancer is either castration alone or in combination with an anti-androgen. This section reviews surgical castration, medical castration and anti-androgens, followed by their use in combination therapy (CAB).

### Surgical castration (orchidectomy)

Bilateral orchidectomy, either total or subcapsular, has been the mainstay of treatment for advanced prostate cancer and is the comparator against which other treatments are assessed. Orchidectomy produces symptom relief in 70–80% of patients (Kaisary et al, 1991), and provides pain relief from symptoms of bone metastases in 80–90% of patients. The size of the prostate tumour shrinks within 4–6 weeks of orchidectomy (Paulson, 1981). Because testosterone levels are reduced so quickly, orchidectomy is often the best treatment for men with metastases in the spine who are at severe risk of paralysis (Korman, 1989). If a surgical option

*Received 16 May 1997*
*Revised 7 May 1998*
*Accepted 19 May 1998*

*Correspondence to:* CJ Tyrrell

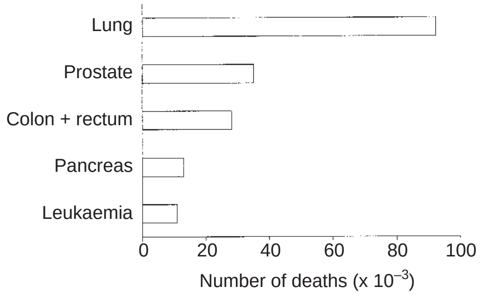

**Figure 1** Reported cancer deaths in men (USA, 1993, from Parker et al, 1997)

is not possible, then ketoconazole can be used to lower testosterone levels very rapidly (Lowe and Bamberger, 1990).

Surgical castration is a relatively simple, safe and inexpensive operation which can be performed under local or light general anaesthesia (Geller et al, 1988; Griffiths et al 1993). Although the convenience of a 'one-off' procedure, as opposed to medical therapy, means patient compliance is not a problem, surgical castration is not acceptable to all patients (Catalona, 1994), mainly because of psychological trauma (Cassileth et al, 1992; Denis, 1993; Fossa et al, 1994). The trauma of castration can be avoided to some extent by use of the subcapsular technique which removes only the functional part of the testicle.

Disadvantages of orchidectomy include loss of libido, impotence and hot flushes (Varenhorst, 1993), which occur in around 60% of men. A further disadvantage is that the operation is irreversible. Patients with non-hormone responsive prostate cancer may, therefore, have undergone unnecessary surgery. If intermittent androgen blockade proves beneficial (see later), then orchidectomy may not be the best treatment option because of its irreversibility.

Orchidectomy reduces circulating testosterone by around 90% (Labrie et al, 1985). However, the intraprostatic concentration of the active androgen, dihydrotestosterone (DHT), after castration is less affected and may amount to 30–40% of normal levels (Labrie et al, 1987; Geller et al, 1988). This residue must be derived from adrenal androgens which make a sizeable contribution to androgen metabolism within the prostate gland.

### Medical castration

### *Luteinizing hormone-releasing hormone (LH-RH) analogues*

LH-RH analogues provide one method of medical castration and are a widely used alternative to surgical castration. LH-RH, produced by the hypothalamus, stimulates production of

**Table 1** Hormone therapies for advanced prostate cancer: advantages and disadvantages

| Treatment | Advantages | Disadvantages |
|---|---|---|
| Orchidectomy | Symptom relief<br>Rapid reduction in circulating testosterone<br>No compliance problems | Does not eliminate adrenal androgens<br>Psychological trauma<br>Irreversible<br>Loss of libido<br>Impotence |
| Medical castration | | |
| Luteinizing hormone-releasing hormone (LH-RH) analogues (e.g. leuprolide, goserelin acetate, buserelin) | As effective as orchidectomy without surgery<br>Reversible<br>Low risk of cardiovascular side-effects<br>Longer acting formulations, e.g. goserelin acetate 12-week depot | Does not eliminate adrenal androgens<br>Risk of tumour flare<br>Loss of libido<br>Impotence |
| Diethylstilboestrol (DES) | As effective as orchidectomy, without surgery | Risk of cardiovascular complications<br>Loss of libido<br>Gynaecomastia<br>Nausea |
| Anti-androgens | | |
| Steroidal (e.g. cyproterone acetate) | Avoids surgery<br>As effective as oestrogens | Loss of libido<br>Impotence<br>Disturbances in liver function<br>Thromboembolism<br>Steroidal effects, e.g. fluid retention |
| Non-steroidal (e.g. flutamide, bicalutamide, nilutamide) | Blocks action of dihydrotestosterone and testosterone<br>Reduces risk of testosterone flare<br>Avoids surgery<br>Most commonly used in combination with surgical or medical castration (CAB)<br>Less cardiovascular toxicity than DES<br>Preservation of potency in 75% of men | Diarrhoea (incidence with flutamide twice that with bicalutamide)<br>Liver toxicity (flutamide)<br>Visual problems (nilutamide only)<br>Alcohol intolerance (nilutamide only)<br>Gynaecomastia<br>Hot flushes |

© Cancer Research Campaign 1999

luteinizing hormone (LH) from the pituitary gland. Testosterone is produced by the testes in response to LH. Negative feedback occurs via the rise in testosterone levels, which brings about a decrease in hypothalamic release of LH-RH. The continuous infusion of LH-RH analogues renders the pituitary refractory to hypothalamic regulation, thus suppressing the release of androgen from the testes. Goserelin acetate, buserelin, leuprolide and triptorelin have all been administered as LH-RH analogues (e.g. Labrie et al, 1985; Crawford et al, 1989). All have similar modes of action and efficacy and plasma testosterone levels are reduced to castrate levels within 2–4 weeks of starting the treatment. Both leuprolide and goserelin acetate have been shown to be as effective as diethylstilboestrol (DES), with objective responses of 50–85% for DES and 70–86% for the LH-RH analogues (Leuprolide Study Group, 1984; Emtage et al, 1988). However, goserelin acetate has superior tolerability to DES (Emtage et al, 1988). Only goserelin acetate has been shown, in major comparative studies, to be equivalent to surgical castration on the basis of the degree of serum testosterone suppression, objective response rates (71% vs 72%), duration of response (53.7 vs 50.1 weeks) and survival (27.5 vs 24.8 months; Debruyne et al, 1988; Kaisary et al, 1991). In a comparison of the effects of surgical castration and goserelin acetate treatment on patients' quality of life (QOL), a significant improvement in two scores of QOL were observed in patients treated with goserelin acetate, but not in those who had surgical castration (Cassileth et al, 1992).

A variety of routes of administration are available for LH-RH analogues and ease of administration may be the deciding factor in the choice of agent. Buserelin is given initially by subcutaneous injection three times daily, then intranasally six times daily, with obvious problems of compliance. The inconvenience of daily injections and the uncertainty of intranasal administration has been overcome by the introduction of biodegradable depot formulations which provide controlled release of LH-RH analogue over a prolonged period. Goserelin acetate, leuprolide and triptorelin are all available as monthly intramuscular or subcutaneous injections. Goserelin acetate is now also available in a 12-weekly depot preparation for subcutaneous injection (Dijkman et al, 1995; Debruyne et al, 1996*a*), and leuprolide is also available as a 3-month depot in some countries (Fernandez Del Moral et al, 1996). These controlled-release preparations have obvious advantages in terms of patient compliance and acceptability.

As expected of an LH-RH agonist, the initial administration may cause a temporary rise in testosterone, which may account for the worsening of symptoms, particularly bone pain, seen in up to 5% of patients (Brewster and Gillatt, 1993; Bruchovsky et al, 1993; Dijkman et al, 1995). This flare phenomenon can have potentially serious effects in patients with spinal secondaries, precipitating spinal cord compression and resulting in paraplegia. Treatment with an anti-androgen 7–10 days before, or concomitantly with, the first injection of LH-RH analogue can prevent the surge of serum testosterone and control the exacerbation of symptoms (Boccon-Gibod et al, 1986; Kuhn et al, 1989; Tyrell et al, 1991). With continued LH-RH analogue treatment, serum testosterone falls to castrate levels and no rise is seen with subsequent injections (Bruchovsky et al, 1993; Brogden and Faulds, 1995).

LH-RH analogues are generally well tolerated: the main side-effects are similar to surgical castration, i.e. loss of libido, impotence and hot flushes (Varenhorst, 1993). Libido and impotence occur in most men treated with LH-RH analogues or surgical castration, whereas hot flushes occur in about 60% of patients (Kaisary et al, 1991; Denis et al, 1993).

### *Oestrogens*

For many years, DES was the only hormonal alternative to orchidectomy. Oestrogens produce their effect partly by suppressing the secretion of LH-RH from the hypothalamus, thereby inhibiting the release of LH from the pituitary, resulting in castrate levels of testosterone, and partly by directly opposing the action of androgens on prostate cells. However, the use of high-dose oestrogens was associated with significant mortality and morbidity because of cardiovascular complications (in up to 25% of patients), including increased incidence of thromboembolism and fluid retention (Veterans Administration Co-operative Urological Research Group, 1967; Allvizatos and Oosterhof, 1993). This led to their use in the treatment of prostate cancer being greatly reduced in the 1970s.

In Scandinavia, oestrogens are still an acceptable therapy for prostate cancer; the drugs commonly used are estramustine phosphate or ethinyl oestradiol in combination with polyestradiol phosphate (Henriksson and Edhag, 1986; Lundgren et al, 1986, 1995). As a primary treatment, estramustine phosphate is reported to be as effective as conventional antineoplastic agents in the treatment of advanced prostate cancer (Perry and McTavish, 1995). As a second-line treatment, estramustine phosphate is no more effective after bilateral orchidectomy than placebo (Iversen et al, 1997*a*; Janknegt et al, 1997).

Recent investigations using high-dose intramuscular-depot oestrogen (estradurin) indicate that cardiovascular side-effects may be lower with this method of administration than with oral administration (Stege et al, 1995). In addition, parenteral administration of polyestradiol phosphate may have bone preserving capacity in patients with prostate cancer (Carlstrom et al, 1997). Further studies by the Scandinavian Prostatic Cancer Group are ongoing and results from a study (SPCG 5) involving over 900 patients on the efficacy and tolerability of parenterally administered polyestradiol phosphate compared with decapeptyl plus flutamide are due to be analysed in 1998.

Recently, there has been increased interest in the use of low-dose oestrogens. Low-dose DES (1 mg day$^{-1}$) was found to be as effective as orchidectomy and associated with fewer malignant disease-related deaths than the more conventional higher dose (3 mg or more). However, it was associated with slightly more deaths (16 out of 108 patients) due to cardiovascular causes than orchidectomy (9 out of 108 patients) (Robinson, 1993). If the risk of cardiovascular toxicity could be controlled, then oestrogens may become a more acceptable option in the management of prostate cancer.

### *Other*

Used in high doses, ketoconazole causes castrate levels of testosterone within 24–48 h and, therefore, has been assessed to determine its role in the treatment of advanced prostate cancer (Lowe and Bamberger, 1990). One clear indication for its use is for treatment of men with metastases of the spine who require a prompt therapeutic response (Bamberger and Lowe, 1988). Other indications include: when orchidectomy is contraindicated, when oestrogens are contraindicated, initial empirical therapy, and hormonally refractory disease. It can also be used in conjunction with LH-RH analogues. However, ketoconazole can cause liver toxicity and

 *© Cancer Research Campaign 1999*

adrenal suppression, although hydrocortisone may be used to minimize toxicity (Small et al, 1997*a*, 1997*b*). Ketoconazole is useful for short-term treatment, but is not particularly useful for long-term therapy (Lowe and Bamberger, 1990).

### Anti-androgens

Anti-androgens act by competitively blocking the binding of testosterone, and its metabolite DHT, to nuclear receptors in prostate cancer cells (Neumann and Jacobi, 1982) and may be steroidal or non-steroidal.

The main steroidal anti-androgen cyproterone acetate (CPA) has been used as oral monotherapy in advanced prostate cancer. This drug reduces testosterone to near castrate levels by its progestogenic effect, suppressing LH-RH and LH. CPA is typically given in a dosage of 200–300 mg day$^{-1}$ in two or three divided doses, and has been shown to be as effective as oestrogen in terms of objective response (40% vs 55%), rate of progression (52% vs 47%) and overall survival (Pavone-Macaluso et al, 1986). Equivalence with surgical castration or LH-RH analogues has not been demonstrated. CPA reduces libido and potency in around 86% of men, a similar incidence to that of surgical and medical castration (Barradell and Faulds, 1994). Other side-effects of CPA include changes in body weight, fatigue, disturbances in liver function (Ohri et al, 1991; Drakos et al, 1992; Watanabe et al, 1994) and thromboembolism (Barradell and Faulds, 1994). CPA has also been evaluated in combined therapy (refer to CAB section).

Non-steroidal anti-androgens, such as nilutamide, flutamide and bicalutamide, have been evaluated for both monotherapy (discussed here) and for combined therapy (discussed in the CAB section) in patients with advanced prostate cancer. The efficacy of nilutamide and flutamide as monotherapy has only been investigated in small non-comparative studies. Only one study showed equal mean time to progression between monotherapy and orchidectomy; in a comparison of flutamide (250 mg three times daily) and orchidectomy involving 104 patients, at the 24-month follow-up, mean time to progression was similar in each group (320 vs 352 days, $P = 0.49$; Boccon-Gibod et al, 1994).

Bicalutamide monotherapy has been evaluated in much larger trials than those carried out for other anti-androgens. Such studies, therefore, are more likely to show up small differences between treatments. A combined analysis of more than 1000 patients showed that bicalutamide monotherapy (50 mg once daily) had a higher treatment failure rate (53% vs 41%), higher objective progression (46% vs 35%) and lower survival (25 vs 28 months, $P = 0.0001$) compared with castration (Bales and Chodak, 1996) demonstrating that although bicalutamide is an effective anti-androgen, it is not equivalent to castration at a dose of 50 mg. Although statistically significant, the clinical significance is open to interpretation. In addition, in two of these trials the survival difference was not significant.

A combined analysis of two multicentre randomized trials comparing bicalutamide 150 mg once daily with castration (goserelin acetate or orchidectomy) showed a survival benefit for castration in metastatic patients, but the survival difference was only 6 weeks (Tyrrell et al, 1996). In addition, the dose of 150 mg had an identical tolerability to the 50-mg dose. In patients with non-metastatic disease (M0), preliminary results suggest that bicalutamide 150 mg may prove equivalent to castration in terms of survival (Tyrrell et al, 1996; Iversen et al, 1997*b*).

When the patient's QOL, tolerability of treatment and sexual function are considered, bicalutamide, either 50 mg or 150 mg, provides significantly better symptom relief, a better QOL and greater preservation of sexual interest compared with castration (Bales and Chodak, 1996; Tyrrell et al, 1996). Higher doses of bicalutamide for use as monotherapy are currently being investigated (Kaisary, 1997).

The non-steroidal anti-androgens obviously do not have the steroidal events associated with CPA. For non-steroidal anti-androgens used as monotherapy, loss of libido and potency is reported in only 20–30% of men (Decensi et al, 1991; Kaisary, 1994), compared with 86% for CPA. As a class, non-steroidal anti-androgens are associated with side-effects such as gynaecomastia (around 40–62% of patients affected), hot flushes (23–50%) and breast pain (26–63%), however there are differences in the side-effect profiles of flutamide, nilutamide and bicalutamide which are unrelated to their anti-androgenic properties.

Nilutamide is associated with a high incidence (20%) of reversible visual abnormalities (Boccardo et al, 1991; Decensi et al, 1991). Approximately one-fifth of patients treated with nilutamide experience alcohol intolerance (Decensi et al, 1991), a problem not reported with any other anti-androgens. Reversible pulmonary interstitial lung disease has been reported with an incidence of approximately 1% in patients treated with nilutamide (Pfitzenmeyer et al, 1992). Nilutamide is not currently available in the UK.

Flutamide monotherapy is associated with a much higher incidence of diarrhoea (29%, Narayan et al, 1996; 20%, Delare and Van Thillo, 1991; 9%, Chang et al, 1996) than bicalutamide monotherapy (2.5%, Kaisary, 1994; 1.9%, Lunglmayr and the International Casodex Study Group, 1995). Raised liver enzymes have been noted in up to 32% of patients after flutamide treatment (Lundgren, 1987). Serious hepatotoxicity has been reported at an annual rate of 3 per 10 000 flutamide users (Wysowski et al, 1993, Wysowski and Foureroy, 1996). Of 19 cases of serious hepatotoxicity reported to the US Food and Drug Administration (FDA) over a 3-year period, five died of progressive liver disease (Wysowski et al, 1993).

Bicalutamide is associated with a lower incidence of side-effects than other anti-androgens. In clinical studies, the incidence of adverse hepatic events, such as raised liver enzymes, during bicalutamide therapy is low (Tyrrell, 1992; Kaisary et al, 1996), and to date there have been no reports of fatal hepatic adverse effects of bicalutamide (Kolvenbag and Blackledge, 1996). Used in combination therapy, bicalutamide was associated with significantly less diarrhoea than flutamide (10% vs 24%; see CAB section; Schellhammer et al, 1996*a*).

### Combined androgen blockade (CAB)

Although LH-RH analogues offer a more acceptable method of castration than surgery, they offer no advantage over orchidectomy in terms of prognosis. This is because the effect of both LH-RH analogues and orchidectomy is limited to blocking production of testicular androgens. Addition of anti-androgens, which block the action of androgens of testicular and adrenal origin, to medical or surgical castration was developed to provide additional androgen blockade (CAB) and so prolong survival of patients with advanced prostate cancer. Bracci and colleagues were the first to utilize CAB, combining CPA treatment with bilateral orchidectomy (Bracci and

© Cancer Research Campaign 1999 

**Table 2** Large randomized trials of combined androgen blockade (CAB)

| Anti-androgen/ investigator | Number of patients | Trial design | Time to progression | Median survival | Follow-up (months) |
|---|---|---|---|---|---|
| Flutamide | | | | | |
| Crawford et al (1997) | 1387 | Orchidectomy + flutamide vs orchidectomy + placebo | NS | NS | 57 |
| Crawford et al (1989, 1995) | 603 | Leuprolide + flutamide vs leuprolide + placebo | CAB superior $P = 0.039$ | CAB superior $P = 0.035$ | 42 |
| Tyrrell et al (1991) | 571 | Goserelin + flutamide vs goserelin | NS | NS | 24 |
| Boccardo et al (1993) | 373 | Goserelin + flutamide vs goserelin | NS | NS | 24 |
| Denis et al (1993) | 327 | Goserelin + flutamide vs orchidectomy | CAB superior $P = 0.008$ | CAB superior $P = 0.02$ | 60 |
| Iversen et al (1993) | 262 | Goserelin + flutamide vs orchidectomy | NS | NS | 57 |
| Fourcade et al (1993) | 245 | Goserelin + flutamide vs goserelin + placebo | NS | NS | 48 |
| Nilutamide | | | | | |
| Janknegt et al (1993, 1996) | 457 | Orchidectomy + nilutamide vs orchidectomy + placebo | CAB superior $P = 0.005$ | CAB superior $P = 0.041$ | 76–102 |
| Crawford et al (1990) | 333 | Leuprolide + nilutamide vs leuprolide + placebo | NS | NS | NR |
| Cyproterone acetate | | | | | |
| Brewster et al (1992) | 349 | Goserelin + CPA vs goserelin | NS | NS | NR |
| Di Silverio et al (1990) | 328 | Goserelin + CPA vs goserelin | NS | NS | 9–58 |
| De Voogt et al (1990) | 307 | Buserelin + CPA vs orchidectomy | NS | NS | 12 |
| Robinson (1993) | 221 | Orchidectomy + CPA vs orchidectomy | NS | NS | 48 |

NS, no significant difference; NR, data not reported; goserelin, goserelin acetate.

De Silverio, 1977; Bracci, 1979). Clinical trials suggesting the efficacy of CAB using flutamide and leuprolide were first reported by Labrie and co-workers (Labrie et al 1982, 1987).

CAB has now been compared with castration alone (medical and surgical) in numerous clinical trials. Some trials show advantage of CAB over castration whereas others report no significant difference (Table 2). No study has reported that CAB is less effective than castration. There is, therefore, considerable debate about the benefits of CAB over castration alone.

Three large, randomized, double-blind, controlled trials comparing CAB with castration have demonstrated a statistically significant improvement for CAB in time to progression and length of survival (Crawford et al, 1989; Denis et al, 1993; Janknegt et al, 1993, 1996). The largest of these trials (603 patients) compared CAB using daily leuprolide and flutamide with leuprolide treatment alone (Crawford et al, 1989). Patients treated with CAB had a longer progression-free survival (16.5 vs 13.9 months, $P = 0.039$) than patients treated with the LH-RH analogue alone. Their median length of survival was also significantly longer (35.6 vs 28.3 months, $P = 0.035$). A subsequent European Organization for Research and Treatment of Cancer (EORTC) trial including 327 patients compared CAB using goserelin acetate plus flutamide with surgical castration (Denis et al, 1993). This trial also demonstrated a statistically significant advantage in favour of CAB for time to progression (33.3 vs 21.3 months, $P = 0.008$) and survival (34.4 vs 27.1 months, $P = 0.02$). In a comparison of orchidectomy plus nilutamide with orchidectomy alone involving 457 patients (Janknegt et al, 1993), a significant ($P < 0.05$) 7-month increase in median survival, before death from cancer, and a progression-free survival advantage of 5.9 months were observed. Long-term follow-up (up to 8 years) also indicated significant benefits in survival and progression-free survival (Janknegt et al, 1996). Additional studies are in progress.

A number of studies have reported equivalence for CAB and castration. Six studies of flutamide in combination with LH-RH analogue compared with LH-RH analogue or orchidectomy alone, with patient numbers of 50–571, showed median times to progression of 16–32 months and median survival of 23–36 months with no statistically significant differences between the treatment arms (Jurincic et al, 1991; Tyrrell et al, 1991; Boccardo et al, 1993; Ferrari et al, 1993; Fourcade et al, 1993; Iversen et al, 1993). Crawford et al (1997) have recently reported results from a prospective, randomized trial comparing flutamide plus orchidectomy with placebo plus orchidectomy in 1387 patients with stage D2 prostate cancer. No statistically significant differences between the groups were found with respect to either time to progression (mean 21 and 18 months respectively) or survival (mean 31 and 30 months). Similarly, studies with nilutamide in combination with either orchidectomy or LH-RH analogue showed no statistically significant difference in time to progression and median survival from the castration alone treatment (medical or surgical; Brisset et al, 1987; Knönagel et al, 1989; Béland et al, 1990; Crawford et al, 1990; Le Duc et al, 1990; Namer et al, 1990). CPA has also been evaluated in combination therapy and there was no significant difference in survival between CPA plus goserelin acetate and the LH-RH analogue alone (Di Silverio et al, 1990; Brewster et al, 1992). With the exception of the recently reported large study by Crawford et al (1997), the individual trials reported above generally have small numbers of patients and, therefore, do not provide sufficient statistical power to demonstrate effect or to statistically refute the results of the three large positive trials (Trachtenberg, 1997). To determine a significant survival benefit in favour of CAB and to ensure treatment groups are balanced, it has been estimated

© Cancer Research Campaign 1999

that it is necessary to include at least 300 patients per treatment arm (Van Tinteren and Dalesio, 1993). The lack of a sufficiently long follow-up period or even a lack of a clear end point, such as survival, were also important factors (Denis, 1995; Trachtenberg, 1997). Interim analyses based on too short a follow-up period can result in too few patients being followed to disease progression to make definite conclusions. Meta-analyses, pooling the results of all studies, is one method to overcome some of these problems.

Meta-analyses, however, also differ on whether CAB shows benefit over castration. A meta-analysis of the individual data from 22 randomized, controlled trials involving 5710 patients treated with either castration (medical or surgical) or various forms of CAB showed a non-significant difference in survival (Prostate Cancer Trialist's Collaborative Group, 1995). Five-year survival rates were 26.2% in the CAB group and 22.8% in the conventional therapy group (these rates are currently being re-evaluated). The Ontario Cancer Treatment Practice Guidelines Initiative suggested that there were a number of methodological weaknesses in the above meta-analysis. These included: the absence of an initial protocol document; no detailed description of search strategy and inclusion/exclusion criteria; no assessment of the quality of the trials, particularly unpublished studies; and the inclusion of data on patients with non-metastatic disease. The authors also noted that a statistically significant difference favouring CAB would have been produced if a one-sided *t*-test (testing the hypothesis that CAB is of benefit or neutral), rather than a two-sided test had been used. Consequently, they conducted a sensitivity analysis of the randomized trials (Klotz and Newman, 1996). An analysis based only on published data (20 studies) demonstrated that therapy with CAB was associated with a clear benefit of 2 years additional survival over castration alone. In addition, the meta-analysis of the Prostate Cancer Trialist's Collaborative Group (1995) was criticized for grouping results of trials that used both steroidal and non-steroidal anti-androgens and the use of immature data (Labrie and Crawford, 1995; Quartey, 1995; Waxman and Pandha, 1995).

An earlier meta-analysis of seven randomized, double-blind trials (1191 patients), which compared CAB (orchidectomy plus nilutamide) with orchidectomy plus placebo in patients who had received no previous hormonal treatment, showed significant delay to disease progression in the nilutamide group compared with the placebo group (Bertagna et al, 1994). Nilutamide used in combination therapy resulted in a statistically significant reduction in risk of progression (16%, $P = 0.05$) and a non-significant 10% reduction in the risk of death. In an update of this analysis, after a further 2-year follow-up, the reduction in risk of progression was maintained (17%, $P = 0.031$, Debruyne et al, 1996*b*). The risk of death from cancer was reduced by 16% ($P = 0.053$). A smaller, meta-analysis of selected trials showed a statistically significant benefit for CAB (Caubet et al, 1996).

Some studies reported a high incidence of withdrawal because of the side-effects of flutamide (Boccardo et al, 1990; Tyrrell et al, 1991; Boccon-Gibod et al, 1992). This may have skewed the results in favour of castration alone, particularly in those trials which did not employ a placebo. It is clear, however, that the tolerability and comparative efficacy of the various anti-androgens should be considered when choosing which anti-androgen to use in CAB treatment.

Only one study has compared anti-androgens in the context of CAB. The study compared treatment with bicalutamide plus LH-RH analogue with flutamide plus LH-RH analogue in 813 patients with untreated metastatic (stage D2) prostate cancer (Schellhammer et al, 1996*a*–1996*c*, 1997; Soloway et al, 1996). At the latest follow-up (median duration 160 weeks), there was an improvement in time to treatment failure with bicalutamide plus LH-RH analogue, although this was non-significant. In terms of survival at 160 weeks, 53% of patients in the bicalutamide group died compared with 57% in the flutamide group. The median survival time was 180 weeks for the bicalutamide group compared with 148 weeks for the flutamide group. The incidence of treatment-related diarrhoea was significantly higher in the flutamide group (26% vs 12%, $P < 0.001$) and caused more treatment withdrawals (25) than in the bicalutamide group (two) (Schellhammer et al, 1997). Diarrhoea has been reported with similar incidence in other flutamide CAB studies (Crawford et al, 1989; Tyrrell et al, 1991). The incidence of diarrhoea in the Crawford study, irrespective of relation to therapy, was reported to the FDA Advisory Committee meeting as 23.8% for the flutamide group and 11.2% for placebo (Schellhammer et al, 1996*d*). Episodes of diarrhoea in flutamide-treated patients have been of sufficient intensity to require withdrawal from therapy in 2–10% of patients (Boccardo et al, 1990; Iversen et al, 1990; Tyrell et al, 1991).

Other factors, such as the stage and extent of disease, can also influence the outcome of CAB treatment. The study by Crawford et al (1989) shows that patients with minimal metastatic disease (defined as five or fewer hotspots on bone scan) and good performance status, who received combined therapy of leuprolide and flutamide, had improved median time to progression compared with patients who received LH-RH analogue alone (48 months vs 19.1 months). Their overall survival was lengthened by 20 months (Crawford et al, 1995). The EORTC meta-analysis, although it had small subgroups, indicated that patients with fewer than five bone metastases and good performance status tended to benefit most from CAB (Denis et al, 1993).

In summary, the overall results of a large number of studies suggest that CAB is at least equivalent to conventional therapy in terms of time to progression and survival. However, opinion is divided on whether CAB has an additional beneficial effect over conventional therapy. CAB's demonstrated equivalence and strong therapeutic rationale suggest that it could be a primary treatment option in advanced prostate cancer. In the absence of a statistically significant difference in the effectiveness of available anti-androgens in CAB, selection may be based on factors such as tolerability. In this respect, bicalutamide may have benefits over flutamide.

### RELATED TREATMENT OPTIONS

### Intermittent therapy

The proposal that prostate cancer cells adapt to androgen deprivation and grow more rapidly in the presence of androgen blockade has resulted in the development of intermittent therapy. The idea is that the patient is treated with androgen blockade until prostate-specific antigen (PSA) is in the normal range, then treatment is stopped until there is evidence of further tumour development (a rise in PSA) when treatment can be started again. It is hypothesized that after the period of androgen exposure (no anti-androgen therapy) the cells will remain sensitive to androgen deprivation and react a second time to anti-androgens rather than progressing to become hormone insensitive. It may not be necessary, therefore, for all patients with limited disease to stay on therapy indefinitely, as

© Cancer Research Campaign 1999

some patients could have intermittent androgen blockade. Preliminary clinical data indicate that this approach is feasible and may offer benefits in terms of QOL and preservation of sexual function (Goldenberg et al, 1995). A randomized prospective study (by the Southwest Oncology Group, SWOG) and an open, non-randomized study (by the European Organization for Research and Treatment of Cancer, EORTC) are currently under way to assess further the value and feasibility of intermittent therapy.

### Withdrawal

There have been several recent reports of favourable clinical and PSA responses to the withdrawal of non-steroidal anti-androgens in patients with progression of disease after lengthy remission while taking CAB (Dupont et al, 1993; Kelly and Scher, 1993; Scher and Kelly, 1993; Nieh, 1995). Scher and Kelly (1993) found that in patients who relapsed while receiving combination therapy of LH-RH analogue and flutamide, if the anti-androgen alone was stopped, the patients went back into remission, some for as long as 2 years. This was not only subjective remission with a falling PSA, but, in those patients with measurable lesions, there was also evidence of objective remission. This withdrawal response is seen in up to 30% of patients, almost exactly the same proportion of patients who respond to single-agent chemotherapy after relapse on hormone therapy. The mechanism for this withdrawal effect is unknown, but it may be related to the development of cancer cell clones that have mutated to be dependent on the anti-androgen as a substrate.

### ADDITIONAL FACTORS IN SELECTION OF TREATMENT OPTIONS

Patients are increasingly involved in decisions about treatment and physicians need to consider the requirements and preferences of individual patients when evaluating treatment options. Physicians both under- and overestimate their patients' subjective morbidity and impact of symptoms on QOL (Osoba, 1994; Calais da Silva et al, 1996). Improvement of QOL and symptom control has become a major end point in clinical trials of prostate cancer (Fossa, 1996). The following issues are relevant to improved QOL of patients with advanced prostate cancer: bone pain, micturition, sexuality, vitality, hot flushes and gynaecomastia. As these factors may be of equal importance to the patient as length of survival, QOL results will need to be incorporated into the overall evaluation of treatment together with survival and health economic considerations.

In studies of patient choice, 78–86% of patients preferred medical castration with the LH-RH analogue goserelin acetate to orchidectomy (Lunglmayr and Girsh, 1987; Cassileth et al, 1989; Fossa et al, 1994). The main motives for choosing LH-RH analogues were avoidance of surgery (36%), success of treatment (18%) and convenience of drug treatment (10%). The reversibility of treatment with goserelin acetate if ineffective was the primary or secondary reason for choosing the drug for 50% of urologists. The primary reasons for patients choosing surgical castration were the convenience of surgical procedure (32%) and success of treatment (29%).

The stage and grade of the disease and the timing of treatment may also give an indication of the success of a particular treatment option. CAB may be more beneficial in minimal disease patients (Crawford et al, 1989; Denis et al, 1993). Disease progression occurs more rapidly and the chance of developing serious complications is increased in patients who receive delayed hormonal treatment compared with those who receive immediate treatment (Kirk, 1996).

Patient compliance is another factor to be considered in selection of treatment for prostate cancer. Compliance is influenced by factors such as efficacy, tolerability and complexity and convenience of the dosing regimen. Differences between the anti-androgens in respect of dosing regimen and tolerability may result in different rates of compliance (Kaisary, 1996). For example, once-daily dosing regimens are associated with significantly better compliance than regimens involving three or four daily doses (Greenberg, 1984). The long elimination half-life of bicalutamide (approximately 7 days; Cockshott et al, 1990) enables once-daily dosing and offers an advantage over anti-androgens with shorter elimination half-lives and more frequent dosing regimens. The active metabolite of flutamide, hydroxyflutamide, has a half-life of 4.3–6.6 h (Brogden and Clissold, 1989) and the drug requires three times daily dosing. Nilutamide, with a half-life of 23–87 h permits once-daily dosing (Harris et al, 1993).

### CONCLUSIONS

Surgical castration is the main hormone treatment for advanced prostate cancer against which other treatments are assessed, although medical castration is an acceptable alternative. CAB combines the benefits of medical or surgical castration with effective blockade of adrenal androgens. The results of clinical trials so far show that CAB is at least equivalent to conventional therapy in terms of survival and progression-free survival. However, opinion is divided on whether CAB has an additional beneficial effect over conventional therapy in advanced prostate cancer. The choice of components for CAB is dependent on the efficacy and tolerability of the various treatments and patient preference. In terms of choice of anti-androgen, bicalutamide may be associated with a lower incidence of side-effects compared with the other non-steroidal anti-androgens and may offer CAB with a lower risk of discontinuation because of intolerance.

Hormonal agents for treating advanced prostate cancer represent a wide range of treatment options. Physicians and patients need to determine the most appropriate option for a given patient based on factors such as the staging extent of the disease, the patient's performance status and the patient's requirements in terms of QOL and survival.

### REFERENCES

Allvizatos G and Oosterhof GO (1993) Update of hormonal treatment in cancer of the prostate. *Anti Cancer Drugs* **4**: 301–309

Bales GT and Chodak GW (1996) A controlled trial of bicalutamide versus castration in patients with advanced prostate cancer. *Urology* **47** (suppl. 1A): 38–43

Bamberger MH and Lowe FC (1988) Ketoconazole initial management and treatment of metastatic prostate cancer to spine. *Urology* **32**: 301–303

Barradell LB and Faulds D (1994) Cyproterone: a review of its pharmacology and therapeutic efficacy in prostate cancer. *Drugs Ageing* **5**: 59–80

Béland G, Elhilali M, Fradet Y, Laroche B, Ramsey EW, Trachtenberg J, Venner PM and Tewari HD (1990) A controlled trial of castration with and without nilutamide in metastatic prostatic carcinoma. *Cancer* **66**: 1074–1079

Bertagna C, de Gery A, Hucher M, Francois JP and Zanriato J (1994) Efficacy of the combination of nilutamide and orchidectomy in patients with metastatic prostatic cancer. *Br J Urol* **73**: 396–402

Boccardo F, Decensi AU, Guarneri D, Rubagotti A, Oneto F, Martorana G, Giuliani L, Delli-Ponti U, Petracco S, Cortellini P, Ziveri M, Ferraris V, Bruttini GP, Epis R, Comeri G and Gallo G (1990) Zoladex with or without flutamide in the

 *© Cancer Research Campaign 1999*

treatment of locally advanced or metastatic prostate cancer: interim analysis of an ongoing PONCAP study. *Eur Urol* **18** (suppl. 3): 48–53

Boccardo F, Decensi AU, Guarneri D, Martorana G, Fioretto L, Mini E, Pavone-Macaluso M, Giuliani L, Santi L and Periti P (1991) Anandron (RU 23908) in metastatic prostate cancer: preliminary results of a multicentric Italian study. *Cancer Detect Prev* **15**: 501–503

Boccardo F, Pace M, Rubagotti A, Guarneri D, Decensi AU, Oneto F, Martorana G, Giuliani L, Selvaggi F, Battaglia M, Delli-Ponti U, Petracco S, Cortellini P, Ziveri M, Ferraris V, Bruttini GP, Epis R, Comeri G and Gallo G (1993) Goserelin acetate with or without flutamide in the treatment of patients with locally advanced or metastatic prostate cancer. *Eur J Cancer* **29A**: 1088–1093

Boccon-Gibod L, Laudat MH, Dugue MA and Steg A (1986) Cyproterone acetate lead-in prevents initial rise of serum testosterone induced by luteinizing hormone-releasing hormone analogs in the treatment of metastatic carcinoma of the prostate. *Eur Urol* **12**: 400–402

Boccon-Gibod L, Fournier G, Botter P and Mallo C (1992) Flutamide versus orchidectomy in patients with metastatic prostate carcinoma (abstract 818). *J Urol* **147**: 417

Boccon-Gibod L, Fournier G, Bottet P, Marechal JM, Guiter J, Rischmann P, Hubert J, Soret JY, Mangin P and Mallo C (1994) Flutamide versus orchidectomy in patients with metastatic prostate carcinoma. *Eur Assoc Urol* (XIth Congress, Berlin 1994) **13**: abstract 25

Bracci U (1979) Antiandrogens in the treatment of prostatic cancer. *Eur J Urol* **5**: 303–306

Bracci U and De Silverio F (1977) Role of cyproterone acetate in urology. In *Androgens and Antiandrogens*. Martini L and Motta M (eds), pp. 333–339. Raven Press: New York

Brawley OW and Kramer BS (1994) The epidemiology and prevention of prostate cancer. In *Prostate Cancer*. Dawson NA and Vogelzang NJ (eds), pp. 47–64. Wiley-Liss: New York

Brewster SF and Gillatt DA (1993) Advanced prostate cancer: what's new in hormonal manipulation? *Br J Hosp Med* **49**: 710–715

Brewster SF, Gillart DA, Chadwick D and Gingell JC (1992) A randomised controlled trial of the LH-RH analogue Zoladex versus cyproterone acetate versus a combination of the two in metastatic carcinoma of the prostate. In *Proceedings of the 10th Congress of the European Association of Urology*, Genoa, July 1992 Puppo P and Guiliani L (eds), Abstract 312, p. 399 Monduzzi Editore SPA: Bologna

Brisset JM, Bertagna C, Fiet J, de Gery A, Hucher M, Hussen J, Tremblay D and Raynaud J (1987) Total androgen blockade vs orchidectomy in stage D prostate cancer. In *Hormonal Manipulation of Cancer*: *Peptides, Growth Factors and New Anti Steroidal Agents*. Klijn JGM, Paridaens R and Foeckens JA (eds), pp. 17–31. Raven Press: New York

Brogden RN and Clissold SP (1989) Flutamide, a preliminary review of its pharmacodynamic and pharmacokinetic properties and therapeutic efficacy in advanced prostatic cancer. *Drugs* **38**: 185–203

Brogden RN and Faulds D (1995) Goserelin. A review of its pharmacodynamic and pharmacokinetic properties and therapeutic efficacy in prostate cancer. *Drugs Ageing* **6**: 324–343

Bruchovsky N, Goldenberg SL, Akakura K and Rennie PS (1993) Luteinizing hormone-releasing hormone agonists in prostate cancer. *Cancer* **72**: 1685–1691

Calais da Silva F, Fossa SD, Aaronson NK, Serbouti S, Denis L, Casselman J, Whelan P, Hetherington J, Fava C, Richards B, Robinson MR and the members of the EORTC Genito-urinary Tract Cancer Cooperative Trial 30853 (1996) The quality of life in patients with newly diagnosed M1 prostate cancer: experience with EORTC Clinical Trial 30853. *Eur J Cancer* **32A**: 72–77

Carlstrom K, Stege R, Henriksson P, Grande M, Gunnarsson PO and Pousette A (1997) Possible bone-preserving capacity of high-dose intramuscular depot estrogen as compared to orchidectomy in the treatment of patients with prostatic carcinoma. *Prostate* **31**: 193–197

Cassileth BR, Seidmon EJ, Soloway MS, Vogelzang N, Schellhammer P, Hait H and Kennealey G (1989) Patients' choice of the treatment in stage D prostate cancer. *Urology* **33**: 57–62

Cassileth BR, Soloway MS, Vogelzang NJ, Chou JM, Schellhammer PD, Seidmon EJ and Kennealey GT (1992) Quality of life and psychosocial status in stage D prostate cancer. *Qual Life Res* **1**: 323–330

Catalona WJ (1994) Management of cancer of the prostate. *N Engl J Med* **331**: 996–1004

Caubet J-F, Tosteston TD, Dong EW, Naylon E, Ernstoff MS and Ross S (1996) Meta-analysis of published randomised clinical trials for MAB in prostate cancer (abstract 654). *Proc Am Soc Clin Oncol* **15**: 254

Chang A, Yeap B, Davis T, Blum R, Hahn R, Khanna O, Fisher H, Rosenthal J, Witte R, Shinella R and Traump D (1996) Double-blind randomized study of primary hormonal treatment of stage D2 prostate carcinoma: flutamide versus diethylstilbestrol. *J Clin Oncol* **14**: 2250–2253

Cockshott ID, Cooper KJ, Sweetmore DS, Blacklock NJ and Denis L (1990) The pharmacokinetics of Casodex in prostate cancer patients after single and during multiple dosing. *Eur Urol* **18** (suppl. 3): 10–17

Crawford ED, Eisenberg MA, McLeod DG, Dorr FA, Davis MA and Goodman PJ (1989) A controlled trial of leuprolide with and without flutamide in prostatic carcinoma. *N Engl J Med* **321**: 419–424

Crawford ED, Smith JA, Soloway MS, Lange PH, Lync DF, Al-Jaburi A, Bracken RB, Heyden N and Bertagna C (1990) Treatment of stage D2 prostate cancer with leuprolide and anandron compared to leuprolide and placebo. In *Recent Advances in Urological Cancers*: *Diagnosis and Treatment*. Murphy G, Khoury S, Chatelain C and Denis L (eds), pp. 27–29. SCI: Paris

Crawford ED, Hussain M, DeAntoni EP, Thompson IM, Eisenberger MA, Blumenstein B and Coltman CA (1995) Southwest Oncology Group Strategies in prostatic carcinoma. *Semin Surg Oncol* **11**: 60–64

Crawford ED, Eisenberger MA, McLeod DG, Wilding G and Blumenstein BA (1997) Comparison of bilateral orchidectomy with or without flutamide for the treatment of patients with stage $D_2$ adenocarcinoma of the prostate: results of NCI intergroup study 0105 (SWOG and ECOG) (abstract 1311). *J Urol* **157**: 336

Dearnaley DP (1994) Cancer of the prostate. *Br Med J* **308**: 780–784

Debruyne FM, Denis L, Lunglmayr G, Mahler C, Newling DWW, Richards BR, Robinson MRG, Smith PH, Weil EHJ and Whelan P (1988) Long-term therapy with a depot luteinizing hormone-releasing hormone analogue (Zoladex) in patients with advanced prostatic carcinoma. *J Urol* **140**: 775–777

Debruyne FM, Dijkman GA, Lee DC, Witges WP, del Moral F, Karthaus HF, van der Mejden AP, Plasman JW, Pull HC, Kums JJ, Idema JG, Hoefakker JW, Heijbroek RP, Kil PJ and Khoe GS (1996*a*) A new long acting formulation of the luteinizing hormone analogue goserelin: results of studies in prostate cancer. *J Urol* **155**: 1352–1354

Debruyne FM, De Gery A, Hucher M and Godfroid N (1996*b*) Maximum androgen blockade with nilutamide combined with orchidectomy in advanced prostate cancer: an updated metaanalysis of 7 randomised, placebo-controlled trials (1191 patients) (abstract 990). *Eur Urol* **30** (suppl. 2): 264

Decensi AU, Boccardo F, Guarneri D, Positano N, Paoletti MC, Constanini M, Martorani G and Giuliani L for the Italian Prostatic Cancer Project (1991) *J Urol* **146**: 377–381

Delare KP and Van Thillo (1991) Flutamide monotherapy as primary treatment in advanced prostatic carcinoma. *Semin Oncol* **18**: 13–18

Denis L (1993) Prostate cancer. Primary hormonal treatment. *Cancer* **71** (suppl.): 1050–1058

Denis L (1995) Commentary on maximal androgen blockade in prostate cancer: a theory put into practice? *Prostate* **27**: 233–240

Denis LJ, Carneiro De Moura JL, Bono A, Sylvester R, Whelan P, Newling D and Depauw M (1993) Goserelin acetate and flutamide versus bilateral orchidectomy: a phase III EORTC trial (30853). *Urology* **42**: 119–130

De Voogt HJ, Klijn JGM, Stude U, Schröder T, Sylvester R and De Pauw M (1990) Orchidectomy versus buserelin in combination with cyproterone acetate, for 2 weeks or continuously, in the treatment of metastatic prostatic cancer. Preliminary results of EORTC trial 30843. *J Steroid Biochem Mol Biol* **37**: 965–969

Dijkman GA, Debruyne FM, Fernandez del Moral P, Plasman J, Hoefakker J, Idema J and Sykes M (1995) A randomized trial comparing the safety and efficacy of the Zoladex 10.8 mg depot, administered every 12 weeks to that of the Zoladex 3.6 mg depot, administered every 4 weeks, in patients with advanced prostate cancer. *Eur Urol* **27**: 43–46

Di Silverio F, Serio M, D'Eramo G and Sciarra F (1990) Zoladex versus Zoladex plus cyproterone acetate in the treatment of advanced prostatic cancer. A multicentre Italian study. *Eur Urol* **18** (suppl. 6): 21–25

Drakos PE, Gez E and Catane R (1992) Hepatitis due to cyproterone acetate. *Eur J Cancer* **28A**: 1932–1933

Dupont A, Gomez J-L, Cusan L, Koutsilieris M and Labrie F (1993) Response to flutamide withdrawal in advanced prostate cancer in progression under combination therapy. *J Urol* **150**: 908–913

Emtage LA, Trethowan C, Hilton C, Kelly K and Blackledge GR (1988) Interim report of a randomized trial comparing Zoladex 3.6 mg depot with diethylstilbestrol 3 mg/day in advanced prostate cancer. *Am J Clin Oncol* **11** (suppl. 2): 173–175

Fernandez Del Moral P, Dijkman GA, Debruyne FM, Witjes WP and Kolvenbag GJ (1996) Three-month depot of goserelin acetate: clinical efficacy and endocrine profile. Dutch South East Cooperative Oncology Group. *Urology* **48**: 894–900

Ferrari P, Castagnetti G, Ferrari G, Pollastri CA, Tavoni F and Dotti A (1993) Combination treatment in M1 prostate cancer. *Cancer* **72** (suppl. 12): 3880–3885

*© Cancer Research Campaign 1999* 

Fossa SD (1996) Quality of life in prostate cancer: what are the issues and how are they measured? *Eur Urol* **29** (suppl. 2): 121–123

Fossa SD, Aass N and Opjordsmoen S (1994) Assessment of quality of life in patients with prostate cancer. *Semin Oncol* **21**: 657–661

Fourcade RO, Colombel P, Mangin M, Grise P, Sorets J, Coulange C, Cariou G, Coloby P, Rivaille A and Azab M (1993) Zoladex plus flutamide versus Zoladex plus placebo in advanced prostatic carcinoma: extended follow-up of the French multicentre study. In *Proceedings 3rd International Symposium on Recent Advances in Urological Cancer*: *Diagnosis and Treatment*, June 1992, Paris. Murphy D, Khoury S, Chatelain C and Denis L (eds), pp. 102–106. SCI: Paris

Geller J, Albert JD and Vik A (1988) Advantages of total androgen blockade in the treatment of advanced prostate cancer. *Semin Oncol* **15** (suppl. 1): 53–61

Goldenberg SL, Bruchovsky N, Gleave ME, Sullivan LD and Akakura K (1995) Intermittent androgen suppression in the treatment of prostate cancer: a preliminary report. *Urology* **45**: 839–845

Greenberg RN (1984) Overview of patient compliance with medication dosing: a literature review. *Clin Ther* **6**: 592–599

Griffiths K, Eaton CL, Harper ME, Turkes A and Peeling WB (1993) Endocrine aspects of prostate cancer. *Rev Endocr Related Cancer* **42**: 5–22

Harris MG, Coleman SG, Faulds D and Chrisp P (1993) Nilutamide. A review of its pharmacodynamic and pharmacokinetic properties and therapeutic efficacy in prostate cancer. *Drugs Ageing* **3**: 9–25

Henriksson P and Edhag O (1986) Orchidectomy vs oestrogen for prostatic cancer: cardiovascular effects. *Br Med J* **293**: 413–415

Iversen P, Christensen MG, Friis E, Hornbol P, Hvidt V, Iversen HG, Klarskov P, Krarap T, Lund F, Morgensen P, Pedersen T, Rasmussen F, Rose C, Skaarup P and Wolf H (1990) A phase III trial of Zoladex and flutamide versus orchidectomy in the treatment of patients with advanced carcinoma of the prostate. *Cancer* **66** (suppl. 5): 1058

Iversen P, Rasmussen F, Klarskov and Christensen IJ (1993) Long term results of Danish prostatic cancer group trial 86. Goserelin acetate plus flutamide versus orchidectomy in advanced prostatic cancer. *Cancer* **82** (suppl. 12): 3851–3854

Iversen P, Rasmussen F, Asmussen C, Christensen IJ, Eickhoff J, Klarskov P, Larsen E, Mogensen P, Mommsen S and Rosenkilde P (1997*a*) Estramustine phosphate versus placebo as second line treatment after orchiectomy in patients with metastatic prostate cancer: Daproca study 9002. *J Urol* **157**: 929–934

Iversen P, Kaisary AV, Tyrrell C, Anderson J, Baert L, Tammela T, Chamberlain M, Webster A and Blackledge G (1997*b*) A randomised comparison of Casodex 150 mg versus castration in the treatment of advanced prostate cancer (abstract 1098). *Br J Urol* **80** (suppl. 2): 279

Janknegt RA, Abbou CC, Bartoletti R, Bernstein-Hahn L, Bracken B, Brisset J, Calais da Silva F, Chisholm G and Crawford E (1993) Orchidectomy and nilutamide or placebo as treatment of metastatic prostatic cancer in a multinational double-blind randomized trial. *J Urol* **149**: 77–83

Janknegt RA, Maastricht NL, Crawford ED, Debruyne F, Dijkman G, Frick J, Goedhals L, Knönagel H and Venner P (1996) Long-term efficacy and safety of maximal androgen blockade (MAB) with nilutamide (Anandron) and castration in stage D2 prostate cancer (abstract 991). *Eur Urol* **30** (suppl. 2): 264

Janknegt RA, Boon TA, Van der Beek C and Grob P for the Dutch Estracyt Study Group (1997) Combined hormono/chemotherapy as primary treatment for metastatic prostate cancer: a randomised, multicenter study of orchiectomy alone versus orchiectomy plus estramustine phosphate. *Urology* **49**: 411–420

Jurincic C, Horlbeck R and Klippel KF (1991) Combined treatment (goserelin acetate plus flutamide) versus monotherapy (goserelin alone) in advanced prostate cancer: a randomised study. *Semin Oncol* **18** (suppl. 6): 21–25

Kaisary AV (1994) Current clinical studies with a new nonsteroidal antiandrogen, Casodex. *Prostate* **5**: 27–33

Kaisary AV (1996) Compliance with hormonal treatment for prostate cancer. *Br J Hosp Med* **55**: 359–366

Kaisary AV (1997) Antiandrogen monotherapy in the management of advanced prostate cancer. *Eur Urol* **31** (suppl. 2): 14–19

Kaisary AV, Tyrrell CJ, Peeling WB and Griffiths K (1991) Comparison of LH-RH analogue (Zoladex) with orchidectomy in patients with metastatic prostatic carcinoma. *Br J Urol* **67**: 502–508

Kaisary AV, Klarskov P and McKillop D (1996) Absence of hepatic enzyme induction in prostate cancer patients receiving 'Casodex' (bicalutamide). *Anti-Cancer Drugs* **7**: 54–59

Kelly WK and Scher HI (1993) Prostate specific antigen decline and antiandrogen withdrawal: the flutamide withdrawal syndrome. *J Urol* **149**: 607

Kirk D (1996) Prostate cancer working party study group. Hormone therapy in advanced prostate cancer – report of the Medical Research Council 'immediate' versus 'deferred' treatment study (abstract 204). *Br J Urol* **77** (suppl. 1): 52

Klotz L and Newman T (1996) Total androgen blockade for metastatic prostate cancer. *Can J Urol* **3**: 102–105

Knönagel H, Bolle JF, Hering F, Senn E, Hodel Th, Neuenschwander H and Biedermann C (1989) Therapy of metastatic prostate carcinoma by orchidectomy plus anandron versus orchidectomy versus placebo. *Helv Chir Acta* **56**: 343–345

Kolvenbag GJ and Blackledge GR (1996) Worldwide activity and safety of bicalutamide: a summary review. *Urology* **47**: 70–79

Korman LB (1989) Treatment of prostate cancer. *Clin Pharm* **8**: 412–424

Kuhn J-M, Billebaud T, Navratil H, Moulonguet A, Fiet J, Grise P, Louis J-F, Costa P, Husson J-M, Dahan R, Bertagna C and Edelstein R (1989) Prevention of the transient adverse effects of a gonadotrophin-releasing hormone analogue (buserelin) in metastatic prostate carcinoma by administration of an antiandrogen (nilutamide). *N Engl J Med* **321**: 413–418

Labrie F and Crawford D (1995) Anti-androgens in treatment of prostate cancer. Letter. *Lancet* **346**: 1030

Labrie F, Dupont A, Belanger A, Cusan L, Lacoursiere Y, Monfette G, Laberge JG, Emond JP, Fazehas ATA, Raynaud JP and Husson JM (1982) New hormonal therapy in prostatic carcinoma: combined treatment with an LH-RH agonist and an antiandrogen. *Clin Invest Med* **5**: 267–275

Labrie F, Dupont A and Belanger A (1985) A complete androgen blockade for the treatment of prostate cancer. In *Important Advances in Oncology*. De Vita VT, Hellman S and Rosenberg SA (eds), pp. 193–200. JB Lippincott: Philadelphia

Labrie F, Luthy I, Veilleux R, Simard J, Belanger A and Dupont A (1987) New concepts on the androgen sensitivity of prostate cancer. *Prog Clin Biol Res* **243A**: 145–172

Le Duc A, Delchambre J and Cariou G (1990) Comparison of anandron and a placebo given beginning the day after surgical castration to patients with bone pain due to metastases of prostate cancers. *Eur Urol* **18** (suppl. 1): 385

Leuprolide Study Group (1984) Leuprolide versus diethylstilbestrol for metastatic prostatic cancer. *N Engl J Med* **311**: 1281–1286

Lowe FC and Bamberger MH (1990) Indications for the use of ketoconazole in management of metastatic prostate cancer. *Urology* **36**: 541–545

Lundgren R (1987) Flutamide as primary treatment for metastatic prostatic cancer. *Br J Urol* **59**: 156–158

Lundgren R, Sundin T, Colleen S, Lindstedt E, Wadstrom L, Carlsson S, Hellsten S, Pompeius R, Holmquist B and Nilsson T (1986) Cardiovascular complications of estrogen therapy for non-disseminated prostatic carcinoma. A preliminary report from a randomized multicenter study. *Scand J Urol Nephrol* **20**: 101–105

Lundgren R, Nordle O, Josefsson K and the South Sweden Prostate Cancer Study Group (1995) Immediate estrogen or estramustine phosphate therapy versus deferred endocrine treatment in nonmetastatic prostate cancer: a randomised multicenter study with 15 years of followup. *J Urol* **153**: 1580–1586

Lunglmayr G and Girsh E (1987) Patient choice in the treatment of advanced prostate cancer. *R Soc Med Int Congress Symp Ser* **125**: 47–53

Lunglmayr G and the International Casodex Study Group (1995) Efficacy and tolerability of Casodex in patients with advanced prostate cancer. *Anti-Cancer Drugs* **6**: 508–513

Lunglmayr G, Girsh E, Meixner EM, Viehburger G and Bieglmayer C (1988) Effects of long term GnRH analogue treatment on hormone levels and spermatogenesis in patients with carcinoma of the prostate. *Urol Res* **16**: 315–319

Namer M, Toubol J, Caty A, Couette JE, Douchez J, Kerbrat P and Droz JP (1990) A randomised double blind study evaluating anandron associated with orchidectomy in stage D prostate cancer. *J Steroid Biochem Mol Biol* **37**: 909–915

Narayan P, Trachtenberg J, Lepor H, Debruyne FMJ, Tewari A, Stone N, Das S, Jimenez-Cruz JF, Shearer R, Klimberg I, Schellhammer PF and Costello AJ (1996) A dose–response study of the effect of flutamide on benign prostatic hyperplasia: results of a multicenter study. *Urology* **47**: 497–504

Neumann F and Jacobi GH (1982) Antiandrogens in tumour therapy. *Clin Oncol* **1**: 41–66

Nieh PT (1995) Withdrawal phenomenon with the antiandrogen Casodex. *J Urol* **153**: 1070–1073

Ohri SK, Gaer JAR and Keane PF (1991) Hepatocelluar carcinoma and treatment with cyproterone acetate. Case report. *Br J Urol* **67**: 213–221

Osoba D (1994) Lesson learned form measuring health-related quality of life in oncology. *J Clin Oncol* **12**: 608–616

Parker SL, Tong T, Bolden S and Wingo PA (1997) Cancer statistics 1997. *CA Cancer J Clin* **47**: 5–27

Paulson DF (1981) Multimodal therapy of prostatic cancer. *Urology* **17** (suppl. 4): 53–56

Pavone-Macaluso M, de Voogt HJ, Viggiano G, Barasolo E, Lardennois B, De Pauw M and Sylvester R (1986) Comparison of diethylstilbestrol, cyproterone acetate

 *© Cancer Research Campaign 1999*

and medroxyprogesterone acetate in the treatment of advanced prostate cancer. Final analysis of a randomized phase III trial of the EORTC urological group. *J Urol* **136**: 624–631

Perry CM and McTavish D (1995) Estramustine phosphate sodium. A review of its pharmacodynamic and pharmacokinetic properties, and therapeutic efficacy in prostate cancer. *Drugs Ageing* **7**: 49–74

Pfitzenmeyer P, Foucher P, Piard F, Coudert B, Braud ML, Gabez P, Lacroix S, Mabille J-P and Camus P (1992) Nilutamide pneumonitis: a report on eight patients. *Thorax* **47**: 622–627

Prostate Cancer Trialist's Collaborative Group (1995) Maximal androgen blockade in advanced prostate cancer: an overview of 22 randomised trials with 3283 deaths in 5710 patients. *Lancet* **346**: 265–269

Quartey P (1995) Anti-androgens in treatment of prostate cancer. Letter. *Lancet* **346**: 1031

Robinson MRG (1993) A further analysis of European Organisation for Research and Treatment of Cancer protocol 30805: orchidectomy versus orchidectomy plus cyproterone acetate versus low-dose diethylstilbestrol. *Cancer* **72**: 3855–3857

Schellhammer PF, Sharifi R, Block N, Soloway M, Venner P, Patterson L, Sarosdy M, Vogelzang N, Jones J and Kolvenbag G for the Casodex Combination Study Group (1996*a*) Maximal androgen blockade for patients with metastatic prostate cancer: outcome of a controlled trial of bicalutamide (Casodex) versus flutamide, each in combination with luteinizing hormone releasing hormone analogue therapy. *Urology* **47** (suppl. a): 54–60

Schellhammer PF, Sharifi R, Block N, Soloway M, Venner P, Patterson A, Sarosdy M, Vogelzang N, Jones J and Kolvenbag G (1996*b*) A controlled trial of bicalutamide versus flutamide, each in combination with luteinising hormone-releasing hormone analogue therapy, in patients with advanced prostatic carcinoma. Analysis of time to progression. *Cancer* **78**: 2164–2169

Schellhammer PF, Vogelzgang NJ, Sharifi R, Block N, Soloway M, Venner P, Patterson L, Sarosdy MF, Jones JA and Kolvenbag JC (1996*c*) Casodex in combination therapy: updated results of a randomized, double-blind trial in 813 previously untreated metastatic prostate cancer patients comparing the antiandrogens Casodex (bicalutamide) and Eulexin (flutamide) in combination with luteinising hormone releasing hormone analogue therapy (abstract 992). *Eur Urol* **30**: 264

Schellhammer PF, Soloway MS, Vogelzang NJ, Sarosdy MF, Jones JA and Kolvenbag GJCM (1996*d*) Letter. *Urology* **48**: 662–663

Schellhammer PF, Sharifi R, Block N, Soloway M, Venner P, Patterson L, Sarosdy MF, Vogelzang NJ, Schellenger JJ and Kolvenbag JC (1997) Clinical benefits of bicalutamide compared with flutamide in combined androgen blockade for patients with advanced prostatic carcinoma: final report of a double-blind, randomized, multicentre trial. Casodex Combination Study Group. *Urology* **50**: 330–336

Scher HF and Kelly W (1993) Flutamide withdrawal syndrome: its impact on clinical trials in hormone refractory prostate cancer. *J Clin Oncol* **11**: 1566–1572

Small EJ, Baron AD, Fippin L and Apodaca D (1997*a*) Ketoconazole retains activity in advanced prostate cancer patients with progression despite flutamide withdrawal. *J Urol* **157**: 1204–1207

Small EJ, Baron A and Bok R (1997*b*) Simultaneous antiandrogen withdrawal and treatment with ketoconazole and hydrocortisone in patients with advanced prostate carcinoma. *Cancer* **80**: 1755–1759

Soloway MS, Schellhammer PF, Sharifi R, Venner P, Patterson AL, Sarosdy M, Vogelzang N, Jones J and Kolvenbag G for the Casodex Combination Study Group (1996) A controlled trial of Casodex (bicalutamide) vs flutamide, each in combination with luteinising hormone-releasing hormone analogue therapy in patients with advanced prostate cancer. *Eur Urol* **29** (suppl. 2): 105–109

Stege R, Carlstrom K, Hedlund PO, Pousette A, Von Schoultz B and Henriksson P (1995) Intramuscular depot estrogens (Estradurin) in treatment of patients with prostate carcinoma. Historical aspects, mechanism of action, results and current clinical status. *Urologe A* **34**: 398–403

Trachtenberg J (1997) Progress in complete androgen blockade. *Eur Urol* **31** (suppl. 2): 8–10

Tyrrell CJ (1992) Casodex: a pure non-steroidal antiandrogen used as monotherapy in advanced prostate cancer. *Prostate* **4** (suppl.): 97–104

Tyrrell CJ, Altwein JE, Klippel F, Varenhorst E, Lunglmayr G, Boccardo F, Holdaway IM, Haefliger JM, Jordaan JP and Sotarauta M (1991) A multicentre randomized trial comparing the luteinising hormone releasing hormone analogue goserelin acetate alone and with flutamide in the treatment of advanced prostate cancer. *J Urol* **146**: 1321–1326

Tyrrell CJ, Kaisary AV, Iversen P, Anderson JB, Baert L, Tammela T, Chamberlain M, Webster A and Blackledge GR (1996) A randomised comparison of Casodex 150 mg versus castration in the treatment of advanced prostate cancer (abstract 411). In *Proceedings of ASCO 15 May 1996* **15**: 192

Van Tinteren H and Dalesio O (1993) Systematic overview (metaanalysis) of all randomized trials of treatment of prostate cancer. *Cancer* **72**: 3847–3850

Varenhorst E (1993) Orchidectomy, GnRh analogues and antiandrogens in advanced carcinoma of the prostate. *Laekemedelsverket* **3**: 89–102

Veterans Administration Co-operative Urological Research Group (1967) Treatment and survival of patients with cancer of the prostate. *Surg Gynecol Obstet* **124**: 1011–1017

Watanabe S, Yamasaki S, Tanae A and Hibi I (1994) Three cases of hepatocellular carcinoma among cyproterone users. *Lancet* **334**: 1567–1568

Waxman J and Pandha H (1995) Anti-androgens in treatment of prostate cancer. Letter. *Lancet* **346**: 1030

Wingo PA, Tong T and Bolden S (1997) An adjustment to the 1997 estimate for new prostate cancer cases. *CA Cancer J Clin* **47**: 239–242

Wysowski DK and Fourcroy JL (1996) Flutamide hepatotoxicity. *J Urol* **155**: 209–212

Wysowski DK, Freiman JP, Tourtelot JB and Horton ML (1993) Fatal and nonfatal hepatotoxiciy associated with flutamide. *Ann Intern Med* **118**: 860–864

© Cancer Research Campaign 1999 
